# CT-Based Analysis of Rod Trace Length Changes During Posterior Spinal Correction in Adult Spinal Deformity

**DOI:** 10.3390/jcm15020778

**Published:** 2026-01-18

**Authors:** Takumi Takeuchi, Takafumi Iwasaki, Kaito Jinnai, Yosuke Kawano, Kazumasa Konishi, Masahito Takahashi, Hitoshi Kono, Naobumi Hosogane

**Affiliations:** 1 Department of Orthopedic Surgery, Kyorin University School of Medicine, 6-20-2, Shinkawa, Mitaka-shi 181-8611, Tokyo, Japan; takumi1107@ks.kyorin-u.ac.jp (T.T.); jin19jin19@gmail.com (K.J.); yosuke-kawano@ks.kyorin-u.ac.jp (Y.K.); koniayu@hotmail.co.jp (K.K.); mtaka@ks.kyorin-u.ac.jp (M.T.); 2 Keiyu Orthopedic Hospital, 2267-1, Akouda-cho, Tatebayashi 374-0013, Gunma-ken, Japan; zakio99_t@yahoo.co.jp (T.I.); kouno-h@ku-kai.or.jp (H.K.)

**Keywords:** adult spinal deformity, spinal fusion, spinal instrumentation, mechanical complications, rod length

## Abstract

**Background**: In adult spinal deformity (ASD) surgery, appropriate rod length determination is crucial, as excessive cranial rod length can lead to skin problems, especially in thin elderly patients if proximal junctional kyphosis (PJK) develops. In adolescent idiopathic scoliosis (AIS), correction is primarily performed in the coronal plane, and rod length changes are relatively predictable. Moreover, PJK is uncommon in AIS, making excess rod length rarely a clinical concern. In contrast, ASD correction involves more complex three-dimensional realignment, including restoration of lumbar lordosis (LL), which makes it challenging to predict postoperative changes in rod trace length (RTL). Furthermore, because PJK occurs more frequently in ASD surgery, appropriate rod length selection becomes clinically important. This study aimed to quantitatively evaluate changes in RTL before and after posterior correction. **Method**: Thirty patients with ASD who underwent staged lateral lumbar interbody fusion (LLIF) followed by posterior corrective fusion from T9 to the pelvis were retrospectively analyzed. RTL before posterior correction (Pre-RTL) was estimated from the planned screw insertional point on axial CT after LLIF, and postoperative RTL (Post-RTL) was measured from screw head centers on post-operative CT. LL and Cobb angle were assessed before and after posterior correction. Correlations between RTL change and alignment change were evaluated. **Results**: Postoperative RTL was shortened in all patients, with an average reduction of approximately 16–17 mm. RTL shortening demonstrated significant correlations with LL correction (R = 0.51, *p* = 0.003) and Cobb angle correction (R = 0.70, *p* = 0.00001). Greater shortening of RTL was observed on the convex side in patients with preoperative Cobb angle ≥ 10° (*p* = 0.04). **Conclusions**: Greater coronal deformity, particularly on the convex side, was associated with increased RTL shortening. These findings suggest that routine preparation of excessively long rods may be unnecessary. Consideration of anticipated RTL shortening may help avoid excessive cranial rod length and potentially reduce the risk of skin complications associated with PJK, particularly in thin elderly patients.

## 1. Introduction

Adult spinal deformity (ASD) causes low back pain, neurological symptoms, gait disturbances, and significant limitations in activities of daily living (ADL), leading to a marked deterioration in patients’ quality of life (QOL) [1,2,3]. With the aging of the global population, ASD associated with degenerative spinal changes has become increasingly common, and the number of corrective spinal surgeries has risen worldwide. However, the incidence of postoperative complications has increased in parallel with the growing surgical volume [4,5]. Mechanical complications (MCs), particularly proximal junctional kyphosis (PJK) and rod fracture (RF), occur frequently and often require revision surgery, resulting in deterioration of clinical outcomes and increased healthcare burden [6].

**Figure 1:**
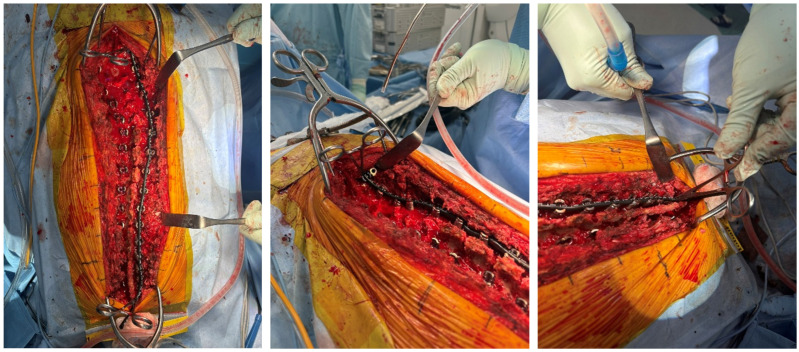
Intraoperative measurement of rod length using a rod template. The rod template is aligned with the screw heads to assess proper rod length before final rod insertion.

Restoring an appropriate LL is crucial for achieving a favorable balance in the sagittal plane [7,8,9,10] and preventing MC after correction surgery for ASD. Because physiological LL must be accurately reproduced in the sagittal plane to achieve optimal clinical outcomes. Both rod contour and rod length play essential roles in achieving this objective. To accomplish this, it is necessary to create a rod with an ideal contour [11,12] and appropriate length during surgery. Typically, a template is used to measure the distance between the upper instrumented vertebra (UIV) and the lower instrumented vertebra (LIV), providing an initial estimate of rod length before final deformity correction (Figure 1). The rod is then cut to a length that accounts for the anticipated changes after correction and subsequently bent into the desired contour. For accurate rod length prediction, both coronal and sagittal plane corrections must be considered. In adolescent idiopathic scoliosis (AIS), rod length changes are relatively predictable because deformity correction is primarily performed in the coronal plane. After correction, the convex side generally becomes slightly shorter, whereas the concave side becomes slightly longer, making rod length estimation comparatively straightforward. In contrast, in ASD with severe kyphoscoliosis, rod length must be determined by accounting for corrections in both coronal and sagittal planes. In particular, substantial lumbar lordosis restoration may require a longer rod due to the increased arc length. However, when combined with coronal realignment, it becomes difficult to predict whether the final rod will ultimately be longer or shorter after three-dimensional correction. Therefore, despite the routine use of templating techniques, accurately predicting changes in rod length during ASD correction remains challenging. If the rod is excessively long, postoperative progression of PJK may lead to cranial rod prominence and result in skin irritation, necessitating intraoperative rod trimming. Conversely, if surgeons anticipate this risk and create a shorter rod than required, replacement with a new, longer rod may become necessary. These issues can prolong operative time, increase soft-tissue dissection, and elevate the risk of further mechanical complications [13].

Although determining the optimal rod length is important in ASD corrective surgery, accurately predicting rod length changes based solely on intraoperative rod template measurements remains difficult, and fundamental knowledge in this field remains limited. Recent advancements such as computer-assisted rod bending [14,15] and robotic surgical planning [16] have improved the accuracy of rod contouring, but these technologies are currently available only in limited institutions and are not widely implemented in routine clinical practice [5,17]. Accordingly, our research question focused on how the distance between pedicle screws, which determines the required rod length, changes during posterior deformity correction in routine clinical practice.

The purpose of this study was to quantitatively evaluate how rod length changes during posterior spinal correction for ASD and to provide fundamental data that may contribute to appropriate rod preparation in ASD surgery.

## 2. Materials and Methods

This study was a retrospective investigation conducted to evaluate changes in rod length during corrective surgery for ASD. To minimize variability due to differences in surgical procedures, we included elderly ASD patients who underwent the same two-stage surgery with identical UIV and LIV levels. Additionally, only cases in which computed tomography (CT) scans were obtained both immediately before and after posterior correction were selected, enabling precise measurement of rod length changes. The subjects were 30 patients (5 males, 25 females; mean age 72.4 years) diagnosed with either degenerative lumbar kyphosis or degenerative lumbar kyphoscoliosis. All patients underwent two-stage ASD surgery with lateral lumbar interbody fusion (LLIF) at L1–5 or L2–5 and posterior fusion from T9 to the pelvis, including L5/S1 posterior lumbar interbody fusion (PLIF) and Ponte osteotomies were applied at all lumbar levels. Since retrospective measurement of the distance from UIV to LIV before correction was not feasible, the distance between pedicle screw (PS) insertion points was measured and defined as the rod trace length (RTL) before correction (pre-RTL). To evaluate changes in RTL during posterior spinal correction, pre-RTL was estimated using CT images obtained after LLIF, whereas post-RTL was estimated using CT images obtained after the second-stage posterior corrective surgery. All measurements were performed using the SYNAPSE VINCENT workstation (Fujifilm Corporation, Tokyo, Japan).

**Figure 2:**
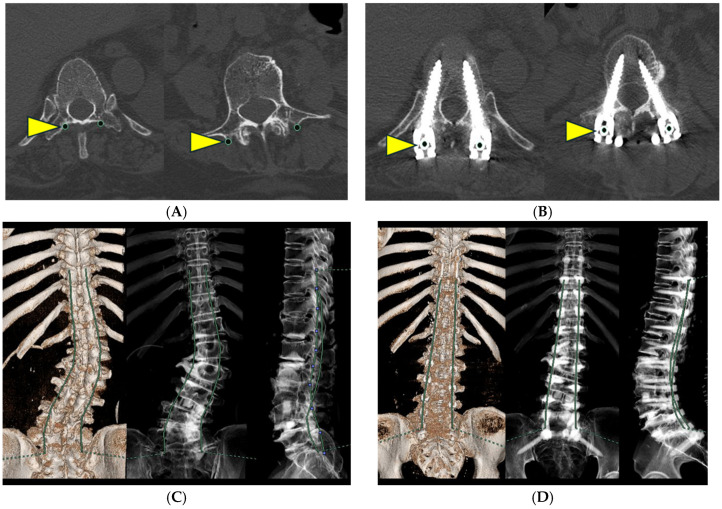
Axial CT images used for plotting rod trace length (RTL) and three-dimensional reconstruction of PSTL from plotted points. (**A**) Planned pedicle screw insertion points were bilaterally plotted on axial CT images obtained after lateral lumbar interbody fusion (LLIF) to calculate the Pre-correction RTL (Pre-RTL). (**B**) Actual pedicle screw head centers were bilaterally plotted on axial CT images obtained after posterior correction to calculate the Post-correction RTL (Post-RTL). (**C**) The RTL before posterior correction (Pre-RTL) was calculated by connecting the planned pedicle screw insertion points from T9 to S1 after lateral lumbar interbody fusion (LLIF). (**D**) The RTL after posterior correction (Post-RTL) was calculated by connecting the actual screw head centers from T9 to S1 after posterior correction. Yellow arrowheads and black circles: plotting points; Green line: Rod trace length (RTL).

**Figure 3:**
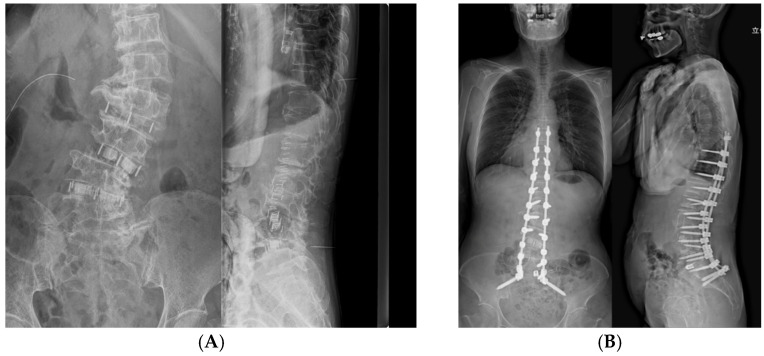
Radiographic measurements of lumbar lordosis (LL) and Cobb angle before and after posterior corrective surgery. (**A**) Anteroposterior and lateral radiographs in prone position in operating room after lateral lumbar interbody fusion (LLIF) for measurements of LL and Cobb angle. (**B**) Standing whole-spine anteroposterior and lateral radiographs taken 4–6 weeks postoperatively for measurement of final alignment.

RTL measurements were limited to the segment between T9 and S1 because the relative distance between the iliac screws and the S1 PS remains essentially unchanged during deformity correction. This approach enabled consistent and reproducible assessment of rod length changes attributable to thoracolumbar deformity correction. For the measurement procedure, pre-RTL was calculated by plotting the planned PS insertion points bilaterally at each vertebral level from T9 to S1 on axial CT images and sequentially connecting these points to reconstruct the rod trajectory, thereby allowing quantitative measurement of rod length (Figure 2A,C). Similarly, post-RTL was calculated by plotting the centers of the PS heads bilaterally at each vertebral level from T9 to S1 on axial CT images after posterior correction and tracing these points (Figure 2B,D). Radiographic measurements were also performed. Before posterior correction, LL and the main thoracolumbar coronal Cobb angle were measured on preoperative prone anteroposterior and lateral X-rays in an operating room (Figure 3A). Postoperatively, LL and Cobb angle were measured from standing whole-spine anteroposterior and lateral X-rays obtained 4–6 weeks after the surgery (Figure 3B). For both CT and plain radiographs measurements, two independent observers performed all measurements twice, and the mean of the two measurements was used for analysis. Inter- and intra-observer reliability was assessed using intraclass correlation coefficients (ICC [2,1]). All measurements were performed twice by two independent observers, and the mean values were used for analysis. The relationship between the RTL changes and the correction angles of LL and Cobb angles was analyzed.

### Statistics

All analyses were performed using EZR (https://www.r-project.org/ accessed on 17 January 2026), which is a modified version of R Commander designed to add statistical functions [18]. All data were statistically analyzed using the two-sample *t*-test for comparisons between convex and concave sides. Pearson’s correlation analysis was used to evaluate the relationship between RTL changes and the correction angles of LL and Cobb angle. The level of significance for all the tests was set at *p* < 0.05.

## 3. Results

**Figure 4:**
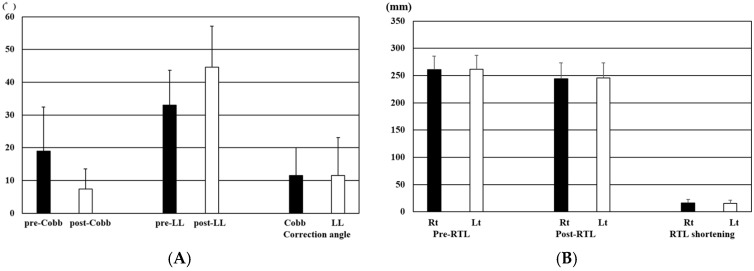
Radiographic changes in alignment and rod trace length (RTL) following corrective surgery; (**A**) Changes in Cobb angle and lumbar lordosis (LL) before and after posterior correction surgery. (**B**) Comparison between RTL before posterior correction (Pre-RTL) and after posterior correction (Post-RTL) on both the right and left sides. The data are shown with mean ± standard deviation.

Inter-observer reliability was excellent for RTL measurements (ICC = 0.93) and good for Cobb angle and lumbar lordosis measurements (ICC = 0.85 and 0.82, respectively). The mean pre- and postoperative Cobb angles were 18.2 ± 11.2° and 6.7 ± 6.3°, respectively, resulting in a mean correction of 11.6 ± 6.9°. The mean LL increased from 31.0 ± 9.8° preoperatively to 45.5 ± 10.8° postoperatively, with an average correction of 14.5 ± 10.3° (Figure 4A) (Table 1).

The mean Pre-RTL was 260.8 ± 24.8 mm on the right side and 261.5 ± 25.3 mm on the left side. The mean Post-RTL was 244.4 ± 28.8 mm on the right side and 245.9 ± 27.1 mm on the left side, resulting in an average reduction of approximately 16mm on both sides (right: 16.5 ± 6.2 mm, left: 15.6 ± 5.8 mm) (Figure 4B) (Table 2).

**Figure 5:**
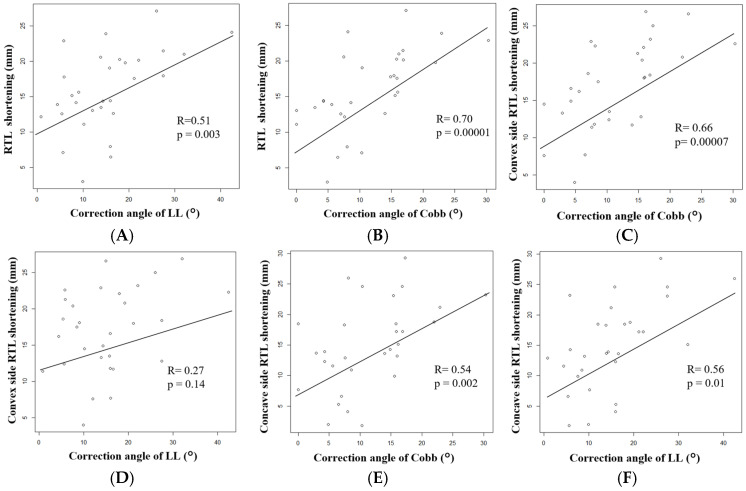
Correlations between rod trace length (RTL) shortening and radiographic alignment correction. (**A**) Overall RTL shortening vs. LL correction. (**B**) Overall RTL shortening vs. coronal Cobb angle correction. (**C**) Convex-side RTL shortening vs. coronal Cobb angle correction. (**D**) Convex-side RTL shortening vs. LL correction. (**E**) Concave-side RTL shortening vs. coronal Cobb angle correction. (**F**) Concave-side RTL shortening vs. LL correction.

Average bilateral RTL shortening showed a significant positive correlation with correction angles of LL (R = 0.51, *p* = 0.003) (Figure 5A) and an even stronger correlation with Cobb angle (R = 0.70, *p* = 0.00001) (Figure 5B).

**Figure 6:**
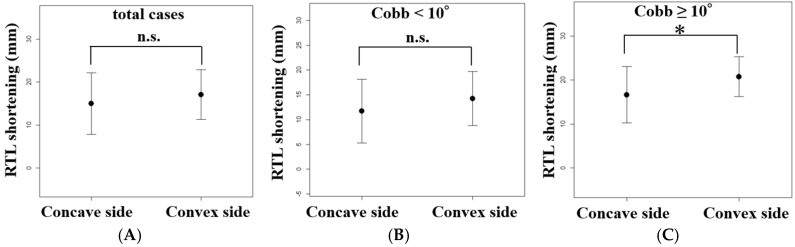
Comparison of rod trace length (RTL) shortening between concave and convex sides after lateral lumbar interbody fusion (LLIF) according to the severity of coronal deformity; (**A**) Overall comparison between convex and concave sides. (**B**) Comparison in patients with Cobb angle < 10°. (**C**) Comparison in patients with Cobb angle ≥ 10°. The data are shown with mean ± standard deviation. *: *p* < 0.05.

Comparison of RTL shortening between the convex and concave sides revealed that the convex side showed a greater reduction (17.1 ± 5.7 mm) than the concave side (15.0 ± 7.1 mm), although the difference was not statistically significant (*p* = 0.21) (Table 2) (Figure 6A).

When analyzed separately for the convex and concave sides, RTL shortening on the convex side demonstrated a positive correlation with the change in Cobb angle (R = 0.66, *p* = 0.00007) (Figure 5C), whereas no significant correlation was observed with the correction angles of LL (R = 0.27, *p* = 0.14) (Figure 5D). In contrast, RTL shortening on the concave side showed positive correlations with both the correction angles of Cobb (R = 0.54, *p* = 0.002) (Figure 5E) and the LL (R = 0.56, *p* = 0.01) (Figure 5F).

To evaluate the impact of the severity of coronal deformity on RTL shortening, patients were divided based on Cobb angle after LLIF (<10° vs. ≥10°). While there was no statistically significant difference in RTL shortening between the concave side (11.7 ± 6.4mm) and the convex side (14.2 ± 5.4 mm) in the Cobb angle < 10° group (n = 14, *p* = 0.27) (Figure 6B), the Cobb angle ≥ 10° group (n = 16) showed a significant difference, with greater shortening on the convex side compared to the concave side (16.7 ± 6.4mm vs. 20.8 ± 4.6 mm, *p* = 0.04) (Figure 6C). Overall, RTL shortened by approximately 16–17 mm in all patients, and the magnitude of shortening showed significant correlations with coronal alignment correction.

### Case Presentation (Case 7)

A 75-year-old female with degenerative lumbar scoliosis underwent two-stage posterior corrective surgery with LLIF. To reduce the risk of PJK, additional tethering was applied at the cranial side of the UIV. Posterior correction and fixation were performed from T9 to the pelvis.

In the prone position after LLIF, the right convex Cobb angle was 36.8° and LL was 34.5°. After posterior correction, standing whole-spine X-rays showed a right convex Cobb angle of 21.9° and LL of 40.4°, indicating corrections of 14.9° and 5.9°, respectively (Figure 3A,B). RTL on the convex (right) side shortened from 267.0 mm to 245.7 mm, representing a reduction of 21.3 mm, whereas the RTL on the concave (left) side decreased from 266.2 mm to 251.9 mm, representing a reduction of 14.3 mm. Thus, RTL shortening of approximately 15–20 mm was observed on both sides after posterior correction (Figure 2C,D; Table 1 and Table 2).

## 4. Discussion

ASD surgery requires not only restoring appropriate LL and global sagittal alignment but also minimizing the risk of postoperative MCs, such as PJK and RF. Previous studies have reported various radiological and surgical risk factors for MCs, including excessive PI–LL mismatch [7], overcorrection [19], poor bone quality [20,21], and rigid UIV fixation [22]. However, their prevention remains challenging due to the multifactorial nature of these complications. Although several predictive scoring systems, such as the GAP score [23], have been proposed, their reliability and reproducibility remain controversial [24,25], and no single strategy has consistently prevented the occurrence of PJK or RF. Recently, new radiological parameters such as the L1 pelvic angle (L1PA) and T4–L1PA mismatch have been reported to improve prediction accuracy and reduce the risk of PJK and RF, but complete prevention remains difficult [26,27].

In addition to creating an ideal rod contour, the use of a rod with an appropriate length is an important but often under-recognized factor in preventing MCs. Excessive cranial rod length may lead to soft-tissue irritation and rod prominence, particularly when PJK progresses postoperatively, whereas insufficient rod length may necessitate intraoperative rod replacement, which requires additional surgical time. Therefore, understanding how rod length changes after three-dimensional deformity correction is essential for both accurate rod preparation and reducing mechanical and soft-tissue–related complications. Since substantial corrections are performed in both the sagittal and coronal planes in ASD, accurately predicting rod length solely from intraoperative templating is challenging. Particularly, when a large amount of LL is restored in the sagittal plane, the actual postoperative rod length often deviates from the preoperative estimate. To address this clinical challenge, the present study quantitatively evaluated intraoperatively measured changes in RTL and clarified their characteristic patterns.

In this study, we quantitatively evaluated changes in RTL during posterior correction in ASD. Postoperative RTL was shortened in all patients, with an average reduction of approximately 16–17 mm. Greater shortening tended to occur on the convex side in cases with larger coronal plane deformity. These observations provide quantitative insight into rod behavior during three-dimensional deformity correction, an aspect that has not been sufficiently examined in previous studies. They further suggest that the final required rod length may actually be shorter than the intraoperatively template-measured length, which contrasts with our clinical expectation that increasing LL during correction would require a slightly longer rod.

From a biomechanical standpoint, predicting changes in RTL in ASD is challenging due to the complex three-dimensional nature of deformity correction, and quantitative data describing how rod length itself changes remain limited in the existing literature. In contrast, in AIS surgery, correction is primarily focused on the coronal plane, resulting in a predictable pattern in which the convex side tends to shorten and the concave side becomes longer after correction, making rod length estimation relatively straightforward. The deformity is generally more flexible, the bone quality is favorable, and pelvic fixation is rarely required. Consequently, the incidence of PJK is much lower in AIS than ASD, and excessive cranial rod length rarely becomes a clinical concern. Therefore, rod length adjustment has not been a major concern in AIS surgery.

From a surgical perspective, appropriate rod length selection represents an important but often under-recognized factor in ASD surgery. Conversely, corrective surgery for ASD requires complex three-dimensional realignment involving both coronal and sagittal plane reconstruction, including restoration of LL and correction of PI–LL mismatch. As a result, RTL may increase or decrease depending on the correction strategy, making preoperative prediction extremely challenging. Moreover, ASD surgery often requires long fusion constructs including pelvic fixation, and the incidence of PJK is significantly higher than in AIS. Therefore, rod length determination represents a clinically important factor directly related to the risk of mechanical complications. In thin, elderly patients—who are common in Japan—limited subcutaneous soft tissue makes cranially protruding rods particularly hazardous, potentially causing skin breakdown or infection if PJK develops. Consequently, excessive rod length should be avoided whenever possible.

In this study, RTL shortening showed significant correlations with both coronal plane (Cobb angle) and sagittal plane (LL) corrections. However, rod shortening on both the concave and convex sides was primarily associated with the magnitude of Cobb angle correction, indicating a dominant influence of coronal plane realignment on change in RTL. Notably, RTL was unexpectedly shortened on both concave and convex sides of the lumbar curve. This finding may be explained by the combined effects of the lumbar and fractional lumbosacral curves. As these two curves are in opposite directions, the concave side of the lumbar curve corresponds to the convex side of the fractional curve, and vice versa, resulting in RTL shortening on both sides.

Because the lumbar curve generally has a longer arc length than the lumbosacral curve, slightly greater shortening may occur on the lumbar convex side. This coronal plane dependent pattern was particularly evident in patients with a Cobb angle ≥ 10°, in whom the difference in RTL shortening between the concave and convex sides was larger, with mean shortening of 16.7 ± 6.4 mm on the concave side and 20.8 ± 4.6 mm on the convex side. In addition, sagittal realignment toward a smoother lordotic curve may also contribute to rod shortening; however, the absence of a significant correlation with LL on the convex side may reflect limited sample size or a weaker association.

Generally, surgeons may select slightly longer rods than intraoperative template measurements to avoid an inadequate short rod that would require repeating the correction procedure with a new rod. However, the present findings suggest that RTL actually shortens during correction in all cases, especially in cases with major coronal deformity, and highlight the risk of overestimating rod length. Thus, preparing longer rods “for safety” should be reconsidered. Although intraoperative trimming of excess rod length is technically feasible, it may increase soft-tissue dissection and prolong operative time, and therefore should ideally be avoided whenever possible. From a clinical perspective, postoperative RTL shortening was observed not only in cases with large residual coronal deformity but also in those with relatively small deformities. Given that the mean RTL shortening in this cohort was approximately 16–17 mm, excessive rod preparation beyond the intraoperatively measured length appears unnecessary in most cases. In patients with large coronal deformity, rod preparation matching the intraoperative measurement before correction may be appropriate, whereas even in cases with smaller deformities, limiting excess rod length to within approximately 10 mm may be sufficient. Notably, the smallest changes in RTL were approximately 2–5 mm in cases with small Cobb angles, supporting this threshold. Recent technological developments, such as semi-automated navigation-guided rod-bending systems [14,15], robotic preoperative simulation [16], AI-based complication prediction models [28], and patient-specific rods (PSRs) [29], have attracted attention as potential solutions for optimizing rod design. However, the widespread adoption of these technologies remains limited due to the need for specialized equipment, infrastructure, and cost. Under current clinical conditions, in which most surgeons still rely on intraoperative rod templating, our results provide useful foundational information. These findings suggest that excessive rod length should be avoided and that rod preparation based on intraoperative measurements may be sufficient.

This study has limitations. It was a retrospective analysis with a relatively small sample size of 30 cases, which may limit statistical power to detect small associations and reduce external validity. Furthermore, RTL was calculated by connecting the centers of PS on CT and was not directly measured intraoperatively before or after deformity correction. As a result, it may not fully reflect actual rod contouring or intraoperative adjustments and may differ from the true rod length used during surgery. Other potentially influential factors, such as rod material, bending technique, and surgeon experience, were not evaluated. Moreover, this study did not include cases involving osteotomy, and global sagittal alignment parameters such as sagittal vertical axis (SVA) and PI–LL mismatch were not evaluated. From a future perspective, further studies should include a larger cohort, incorporate cases with varying deformity severity including osteotomy procedures, and evaluate rod-related factors such as material, diameter, and contouring characteristics. In addition, performing direct intraoperative measurements before and after correction will be essential to validate the accuracy and clinical applicability of the calculated RTL values. Developing a predictive model to estimate final rod length based on preoperative radiological data will be an important direction.

## 5. Conclusions

In two-stage corrective surgery for ASD, RTL was shortened in all cases, with an average of 16 mm after posterior correction. The shortening was greater on the convex side in cases with larger coronal plane deformity. These findings suggest that excessive rod length should be avoided and that rod preparation based on intraoperative measurements may be sufficient. Appropriate rod length selection may also help reduce the risk of skin and soft-tissue complications associated with PJK, particularly in thin elderly patients.

## Figures and Tables

**Table 1:** Radiographic parameters measured before and after surgical correction. Parameters include lumbar lordosis (LL) and coronal Cobb angle, assessed from prone and standing radiographs. The data are presented as mean ± standard deviation. LL: lumbar lordosis; Cobb: coronal Cobb angle; SD: standard deviation.

Case	Age	Sex	Convex Side	Pre-Cobb	Post-Cobb	Correction Angle of Cobb (°)	Pre-LL	Post-LL	Correction Angle of LL (°)
1	75	F	Rt	3	3	0	43	55	12
2	62	M	Lt	16	8.4	7.6	33	33.8	0.8
3	65	F	Rt	42.8	12.5	30.3	54.4	60.2	5.8
4	84	M	Rt	28.4	12.4	16	23.8	32.8	9
5	76	F	Rt	11	5.4	5.6	45.6	50	4.4
6	66	F	Rt	2	2	0	27.3	37.5	10.2
7	75	F	Rt	36.8	21.9	14.9	34.5	40.4	5.9
8	71	F	Lt	9.5	2.5	7	11	16.4	5.4
9	67	F	Lt	37.1	15.1	22	34.4	53.6	19.2
10	71	F	Lt	13.2	2.8	10.4	39.9	55.7	15.8
11	71	F	Lt	35.5	12.6	22.9	31.7	46.7	15
12	71	F	Rt	25.7	8.8	16.9	32.7	54.8	22.1
13	71	F	Rt	9.4	1.3	8.1	20.1	62.5	42.4
14	71	M	Lt	8.7	1.2	7.5	40.3	54.1	13.8
15	71	F	Rt	5.3	1	4.3	24.2	40.1	15.9
16	80	F	Rt	22.6	5.3	17.3	22.5	48.5	26
17	82	F	Rt	20.5	3.7	16.8	16.2	43.7	27.5
18	76	F	Lt	20.4	4.2	16.2	28.1	60.1	32
19	76	F	Lt	33.8	25.2	8.6	43.1	51.6	8.5
20	69	M	Rt	7.5	1	6.5	22	38	16
21	75	F	Rt	9.8	4.9	4.9	22.6	32.5	9.9
22	78	F	Rt	5.2	2.3	2.9	34.1	48	13.9
23	70	F	Rt	9	1	8	20.3	36.2	15.9
24	71	F	Rt	15.9	0.5	15.4	27.7	55.2	27.5
25	68	F	Lt	24.1	10.1	14	26.7	43.3	16.6
26	62	F	Rt	22.6	6.8	15.8	32.9	50.9	18
27	85	F	Rt	5.2	0.9	4.3	49.9	64.2	14.3
28	79	M	Lt	16.5	0.9	15.6	33	40.6	7.6
29	78	F	Lt	22.9	12.6	10.3	30	35.6	5.6
30	56	F	Lt	25.8	9.9	15.9	25.3	46.4	21.1
Mean ± SD	72.4 ± 6.5			18.2 ± 11.2	6.7 ± 6.3	11.6 ± 6.9	31.0 ± 9.8	45.5 ± 10.8	14.5 ± 10.3

**Table 2:** Comparison of rod trace length before and after posterior correction surgery. The rod trace length (RTL) before posterior correction (Pre-RTL) was measured following lateral lumbar interbody fusion (LLIF), while RTL after posterior correction (Post-RTL) was measured following posterior correction. The data include shortening on both right and left sides, and convex and concave sides. The data are presented as mean ± standard deviation. RTL; rod trace length; Rt; right; Lt: left; SD: standard deviation.

Case	Pre-RTL (mm)		Post-RTL (mm)		RTL Shortening (mm)			RTL Shortening (mm)	
	Rt	Lt	Rt	Lt	Rt	Lt	Mean ± SD	Convex	Concave
1	269.7	273.4	262.1	254.9	7.6	18.5	13.05	7.6	18.5
2	283.1	288	270.2	276.6	12.9	11.4	12.15	11.4	12.9
3	253.6	238.3	231	215.1	22.6	23.2	22.9	22.6	23.2
4	250.9	246.1	232.8	232.9	18.1	13.2	15.65	18.1	13.2
5	264	264	247.8	252.4	16.2	11.6	13.9	16.2	11.6
6	237.4	236.8	222.9	229.1	14.5	7.7	11.1	14.5	7.7
7	267	266.2	245.7	251.9	21.3	14.3	17.8	21.3	14.3
8	276.5	283.2	269.9	264.6	6.6	18.6	12.6	18.6	6.6
9	246.4	252.6	227.6	231.8	18.8	20.8	19.8	20.8	18.8
10	254.9	246.7	230.3	233.2	24.6	13.5	19.05	24.6	13.5
11	221.2	233.3	200	206.7	21.2	26.6	23.9	26.6	21.2
12	260.5	253.2	237.3	236	23.2	17.2	20.2	23.2	17.2
13	249	250.8	226.7	224.8	22.3	26	24.15	22.3	26
14	271.5	276.6	253.2	253.7	18.3	22.9	20.6	22.9	18.3
15	272.1	270.3	255.5	258	16.6	12.3	14.45	16.6	12.3
16	238.1	239.1	208.8	214.1	29.3	25.0	27.2	29.3	25.0
17	266.3	266	241.7	247.6	24.6	18.4	21.5	24.6	18.4
18	256.5	249.9	229.6	234.8	26.9	15.1	21.0	26.9	15.1
19	238.5	251.1	227.6	233.6	10.9	17.5	14.2	17.5	10.9
20	264.5	264.7	256.8	259.4	7.7	5.3	6.5	7.7	5.3
21	260	257.7	256	255.7	4.0	2.0	3.0	4.0	2.0
22	262.6	268.4	249.3	254.7	13.3	13.7	13.5	13.3	13.7
23	262.4	253.3	250.6	249.2	11.8	4.1	8.0	11.8	4.1
24	266.2	258.6	243.1	245.8	23.1	12.8	18.0	23.1	12.8
25	251.7	259.6	240	246	11.7	13.6	12.7	13.6	11.7
26	233	235.6	210.9	217.1	22.1	18.5	20.3	22.1	18.5
27	255.4	255	240.5	241.1	14.9	13.9	14.4	14.9	13.9
28	255.1	261.1	245.2	240.7	9.9	20.4	15.2	20.4	9.9
29	373.1	376.7	371.3	364.3	1.8	12.4	7.1	12.4	1.8
30	264.2	268.3	247	250.3	17.2	18.0	17.6	18.0	17.2
Mean ± SD	260.8 ± 24.8	261.5 ± 25.3	244.4 ± 28.8	245.9 ± 27.1	16.5 ± 6.2	15.6 ± 5.8	16.0 ± 5.6	17.1 ± 5.7	15.0 ± 7.1

## Data Availability

The data presented in this study are available from the corresponding author upon reasonable request due to ethical and privacy restrictions.

## Abbreviations

ADL (activities of daily living), AIS (adolescent idiopathic scoliosis), ASD (adult spinal deformity), CARBS (computer-assisted rod bending system), Cobb (coronal Cobb angle), CT (computed tomography), GAP score (Global Alignment and Proportion score), ICC (intraclass correlation coefficient), IRB (institutional review board), LL (lumbar lordosis), LLIF (lateral lumbar interbody fusion), LIV (lower instrumented vertebra), L1PA (L1 pelvic angle), MC (mechanical complication), PJK (proximal junctional kyphosis), PLIF (posterior lumbar interbody fusion), PS (pedicle screw), PSR (patient-specific rod), QOL (quality of life), RF (rod fracture), RTL (rod trace length), SVA (sagittal vertical axis), UIV (upper instrumented vertebra).
